# Towards Deep-Learning-Driven Intrusion Detection for the Internet of Things

**DOI:** 10.3390/s19091977

**Published:** 2019-04-27

**Authors:** Geethapriya Thamilarasu, Shiven Chawla

**Affiliations:** School of STEM, University of Washington Bothell, Bothell, WA 98011, USA; chawls@uw.edu

**Keywords:** Internet of Things (IoT), Intrusion-Detection System (IDS), security, deep learning, machine learning

## Abstract

Cyber-attacks on the Internet of Things (IoT) are growing at an alarming rate as devices, applications, and communication networks are becoming increasingly connected and integrated. When attacks on IoT networks go undetected for longer periods, it affects availability of critical systems for end users, increases the number of data breaches and identity theft, drives up the costs and impacts the revenue. It is imperative to detect attacks on IoT systems in near real time to provide effective security and defense. In this paper, we develop an intelligent intrusion-detection system tailored to the IoT environment. Specifically, we use a deep-learning algorithm to detect malicious traffic in IoT networks. The detection solution provides security as a service and facilitates interoperability between various network communication protocols used in IoT. We evaluate our proposed detection framework using both real-network traces for providing a proof of concept, and using simulation for providing evidence of its scalability. Our experimental results confirm that the proposed intrusion-detection system can detect real-world intrusions effectively.

## 1. Introduction

The Internet of Things (IoT) has emerged as the next big technological revolution in computing in recent years with the potential to transform every sphere of human life. With an expanding network of interconnected Internet-enabled devices, IoT devices are used in a range of applications from connected cars, smart homes, healthcare, smart retail, and supply-chain management [1,2]. Juniper research predicts that nearly 38 billion devices will be connected to the Internet by the year 2020 [3].

The rise of this transformative technology is however deeply mired with security and privacy concerns [4,5,6,7]. The massive influx of connected devices introduces new vulnerabilities and opens new avenues for security attacks. To make things worse, IoT devices are often manufactured with little regard for security and/or shipped without adequate security controls in place. Research shows that a large percentage of current IoT devices on the market have serious security flaws and vulnerabilities [8]. Simple IoT devices can be turned into attack vectors if not properly secured. Recent evidence of this was observed in October 2016 where hacked IoT devices (cameras and DVRs) targeted Dyn, the largest DNS provider, resulting in massive Internet outage, disrupting the availability of popular websites such as Twitter, Amazon, and Netflix [9]. More recently, researchers have been able to cause a car crash in the autopilot mode [10], demonstrating the vulnerabilities of connected vehicles. New trends in cyber-attacks (ransomware, malware) increasingly target connected medical IoT devices resulting in attacks on healthcare institutions, compromised electronic health record systems, or in the worst-case scenario, even loss of life [11].

Even with some security measures in place, IoT networks are still vulnerable to multiple attacks due to their large attack surface. It is hence essential to be able to design defense mechanisms capable of detecting attacks. Security defense measures such as intrusion-detection systems (IDS) are critical to vulnerable IoT environments. Due to intrinsic resource and computational constraints, traditional security methods cannot be directly applied to secure IoT systems. Existing research on intrusion detection for the IoT is largely focused on rule-based detection techniques [12,13,14]. For efficient detection of zero-day threats, anomaly-based detection techniques are essential especially in emerging IoT environments. Also, in an IoT ecosystem, where many devices are constantly generating tons of data, machine-learning algorithms can be useful to perform automated data analysis and provide meaningful interpretations and predictions about the system. Use of machine learning for IoT security is especially very promising to detect any outliers to normal activity in the system. To that end, we explore an anomaly-based IDS that leverages the use of machine-learning techniques, to detect any known or unknown attacks on IoT devices in real time.

Our goal is to develop a secure, portable, and ready-to-deploy security system that provides a practical and effective solution for securing future large-scale IoT networks. In this article, we present an Integrated Intrusion-Detection (IID) system that works independent of IoT protocols and network structure, and requires no prior knowledge of security threats. We develop an artificially intelligent IDS to provide security as a service to IoT networks. We provide an overview of the initial design framework of the proposed system in our earlier research [15]. In this article, we significantly expand the framework to incorporate a deep-learning algorithm to adapt to the changing threat landscape and network topology for anomaly detection. Our approach requires no prior knowledge of captured network payload binaries, traffic signatures, or compromised node address. The proposed IDS categorizes the network traffic into sessions and investigates anomalous characteristics of network activity. The main contributions of this research are:

- Development of an anomaly-based intrusion-detection model using deep learning for IoT networks.
- Implementation and evaluation of the model for efficiency.

The outline of the article is as follows. Section 2 discusses the literature review and the related work. Section 3 describes the proposed methodology and implementation of our IDS, including network monitoring algorithm and anomaly-detection algorithm, and describes how the IID dispatches a mitigation response. Section 4 provides implementation details of the IDS developed to demonstrate a proof of concept. Section 5 shows the results for evaluation of this system on real-time network traffic. Section 6 presents the conclusion and future work.

## 2. Related Work

While the IoT has gained popularity, security, privacy and reliability challenges pose a significant barrier to widespread adoption and deployment of these devices [16,17,18,19]. The security vulnerabilities introduced by heterogeneity and interconnectivity of IoT devices and applications makes them a prime target for cyber-criminals to exploit. The increasing number of attacks and information leaks from the IoT has resulted in a surge of research addressing security and privacy in this domain [5,7,20,21]. Despite several preventive measures in place, IoT systems continue to be targeted by malicious actors. It has become more pertinent than ever to emphasize early detection of intrusions to minimize the negative impact on these systems.

Kasinathan et al. proposed a signature-based IDS for 6LoWPAN-based IoT networks [12]. The goal of this system was to detect DoS attacks with a lower false positives rate. This system was later extended to monitor larger networks [22]. Danda et al. proposed a host-based IDS for IoT that also depends on rule-based detection [23]. Both solutions rely on signature and rule-based detection systems that are not capable of detecting new and unknown attacks in IoT. These solutions also face the challenge of frequently updating the signature database.

Le et al. proposed a specification-based IDS, where a human expert manually defines the rules for each specification, to address RPL attacks such as rank-attack, local repair attack, and the resource-depletion attack [24]. Surendar et al. developed a specification IDS using behavioral rules to detect sinkhole attacks on 6LoWPAN networks [25]. The specification-based systems discussed above are limited in that they are only as effective as the expertise level of the expert system. Incorrect specifications can result in false detections and might jeopardize the network security. Intrusion detection based on protocol model are developed to detect anomalies associated with specific IoT protocols [25,26,27,28]. Raza et al. proposed a hybrid signature and anomaly-based IDS, known as SVELTE, for IoT networks based on 6LoWPAN protocol [28]. This system is however based on a centralized IDS and cannot monitor traffic between local nodes in the network and is specifically targeted only towards detecting routing protocol attacks in IoT. Bostani et al. proposed a hybrid specification IDS for detecting RPL-based protocol attacks [26]. The specification-based anomaly module was used to analyze the behavior of the host nodes while the anomaly-based agent employed the unsupervised optimum-path forest algorithm for projecting clustering models. While this method presented promising results, performance of the proposed framework can be improved by incorporating data mining techniques and machine-learning methods.

Machine-learning techniques have been used to improve the effectiveness of intrusion detection in traditional networks. With the advent of smart attacks on IoT, along with their resource and computation constraints, it is necessary to explore the use of machine learning for securing IoT systems. Liu et al. used machine learning and signature-based model to detect new attacks in IoT [29]. This approach uses artificial immune system for self-adaptation and self-learning to detect new attacks. Even with machine learning, this approach still suffers from the setbacks of a signature-based detection. Krimmling et al. proposed machine learning for anomaly and signature-based intrusion detection for securing IoT networks using the Constrained Application Protocol (CoAP) [13] for transport applications. The authors demonstrated using their own evaluation framework that the attack detection techniques failed when used individually but presented improved performance when signature and anomaly-detection approaches were combined. Arrignton et al. proposed a host-based IDS that uses machine learning for anomaly-based intrusion detection [30]. The behavioral model proposed in this system uses artificial immune systems that increases in complexity with the growth of IoT network, eventually becoming resource-consuming, and degrading system performance. Liu et al. developed an IDS using suppressed fuzzy clustering and PCA algorithms [31]. This approach combined machine learning and data mining techniques and demonstrated better detection efficiency as compared to Bayesian and neural-network algorithms. However, with increase in data volume, efficiency and accuracy of the IDS decreases. The authors also note that new features of IoT will be necessary to improve their detection model.

It is evident from the recent research that machine learning for intrusion detection in the IoT is rapidly growing. However, conventional machine-learning algorithms often present low accuracy and/or less scalability for attack detection on large-scale IoT networks. Despite the existing research efforts, machine learning for anomaly detection is still in its initial stages. This article aims to further this research by specifically investigating deep-learning models for intrusion detection in an IoT environment.

## 3. Intrusion-Detection Framework

To effectively detect emerging cyber-attacks on the IoT, we develop an independent IID system aided by deep-learning algorithms. In this section, we discuss the system architecture and components of the proposed IDS framework.

### 3.1. System Architecture

**Figure 1:**
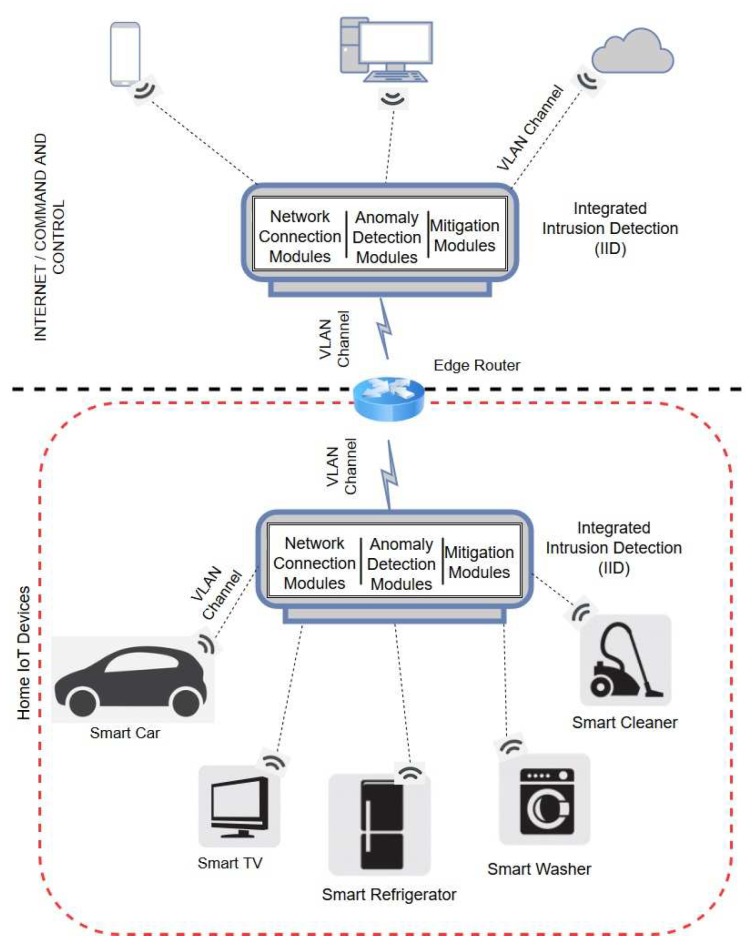
Intrusion Detection System Overview.

Figure 1 shows the IoT network in consideration. IoT devices in the network are portable and geographically distributed within a range defined by the network and the edge router acts as the hub or the coordinator. Devices communicate using wireless communication protocols such as Wi-Fi, Bluetooth BLE, ZigBee, or a proprietary communication protocols such as CoAP or Thread. The proposed IDS is a standalone device that can be easily integrated into the IoT network. The IID works in promiscuous mode, monitoring and analyzing the network traffic, and uses network virtualization to connect to router or other IoT devices.

Below, we briefly outline the different components of our proposed IDS. More details about the IDS framework and its components can be found in our earlier paper [15].

### 3.2. Components of Detection System

**Figure 2:**
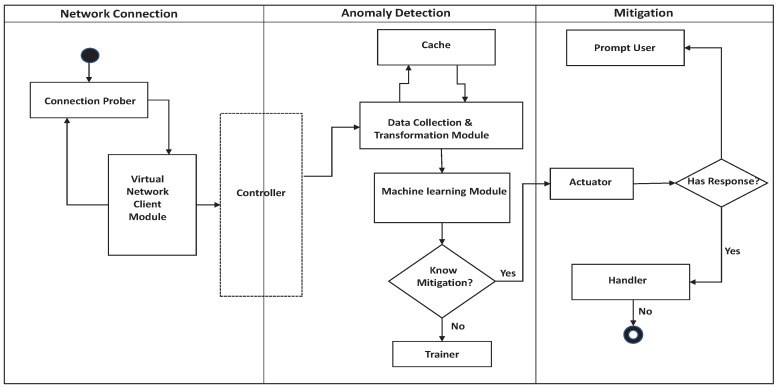
IDS Detection Process.

The proposed IDS functions primarily in three phases, namely the Network Connection Phase, Anomaly-Detection Phase, and the Mitigation Phase as shown in Figure 2.

#### 3.2.1. Network Connection Phase

During the Network Connection Phase, the proposed IDS attempts to determine and deploy appropriate network channel for facilitating translation of sniffed network traffic. The IDS works in transport layer and monitors traffic using two modules in this phase, namely the connection prober module and the virtual network client module.

(i) **Connection Prober:** The connection prober module is responsible for sending probe-signals and broadcast beacons to all the devices within the host IoT personal area network. The module is executed periodically and on-line, as the communication links are constructed dynamically within an IoT network. Connection Prober module maintains a list of all the active communications protocols that are being used in the network. It uses this list to maintain active network interfaces which can be used to intercept different wireless communication signals, transmitted in the surrounding environment. Consequently, it attempts to intercept broadcast beacons, handshake messages, or session requests to learn the communication protocols that are being used by IoT devices. On receiving a handshake or a session request, the connection prober module translates the data-packet into appropriate network packet format. Typically, the connection prober module is designed to use a secondary storage location as a default cache location that is simultaneously replicated in the physical memory using a *cache-handle*. After fetching the bit stream, it uses the known communication protocols to bisect the bit stream into different networks packets and then feeds these packets into cache and the data collection and transformation module (discussed in the next subsection).
  If the connection prober fails at intercepting the broadcast beacons, or handshake messages, it attempts to deduce the connection protocol by apprehending the data packets transmitted during regular data communication of surrounding IoT devices. This is made possible because CoAP communication cycle involves at least 4 successful messages and when connection prober fails to intercept a communication, it attempts to gather information from successive messages, resulting in CP’s probability of success, $P\left(S\right)=\frac{3}{4}$. The number of messages in an IoT communication cycle increases as bare IoT communication is loaded with payload. With increase in number of messages, probability of successful interception increases, resulting in $P\left(S\right)>\frac{3}{4}$, approaching towards unity. In worst-case scenario, if CP misses the entire IoT communication cycle, the IDS uses pre-trained neural networks to estimate the network behavior in the current period. Subsequently, with further training the IDS can be trained to respond to a new protocol.
(ii) **Virtual Network Client (VNC):** The VNC module is a client-based network emulator responsible for establishing compatible network channels with various IoT devices after gathering information about their network protocols. VNC module transforms the packets from different network channels and switches the network protocol according to a specific IoT device.
(iii) **Controller:** The Controller module is responsible for controlling and interfacing the exchange of data packets or commands between the data collection & transformation module and the VNC module. All the modules other than connection prober and VNC module are autonomous and not governed by the Controller module.

#### 3.2.2. Anomaly-Detection Phase

In this phase, data packets are assembled and transformed before feeding into the machine-learning module. Instead of requiring a dedicated host or specialized hardware, this module performs highly optimized data collection and transformation thereby enabling IIDs to be implemented on resource-constrained networks. The components of this phase are described below.

(i) **Data Collection and Transformation (DCT):** DCT module is responsible for stripping the data packets, extracting the header tags as features, populating the cached database, and feeding these tuples into machine-learning-based anomaly-detection module. The features extracted in this work is listed in Table 1. Algorithm 1 details the process where each input network packet is sliced into distinct layers of the TCP/IP stack, and thereafter, respective header tags are extracted for each layer in string format. Each non-empty layer is then designated a label for future reference. Consequently, the extracted header tags are added to a list under the label of their respective layer, thereby removing any repetition of header tags. The data collection and transformation module also pipelines these lists back to the cache.
**Algorithm 1** Extracting tags from the sniffed network packets.**Require:** T - List of all header tags from all packets in network interface queue. **function**
PacketHandler(pkt)         /*where pkt - captured network packet*/  Extract TagString         /*Get tagstring from the packet*/  Divide the TagString for each Layer  **for** every Layer in Packet
**do**   Get LayerName   **if**
Layer≠Empty
**then**    Split the Tags   **end if**   **for** every Tag
**do**    Add the Tag to a list   **end for**   Assign the List to Layer         /*assign list to layer*/  **end for** **end function**
(ii) **Machine-learning-based anomaly-detection (MLAD):** This module is the principal machine-learning engine of the proposed IDS, responsible for classifying benign network traffic from malicious network traffic. It employs a perceptual learning model for performing anomaly detection. This module is activated when the IDS enters the *Anomaly-Detection* phase, which consists of two phases, i.e., the training phase and the detection phase. The training phase is performed across long intervals of time, and performed off-line. The perceptual model is trained using supervised learning over the tuples of features generated during the data-preprocessing. Before feeding the tuple into the perceptual learning model, each tuple is manually augmented with a binary-classification label representing malicious or benign nature of network packet. The perceptual learning model uses information gain at each perceptual layer to filter out the preferred features, before feeding to the next perceptual layer. We discuss the MLAD module in detail in Section 4.
(iii) **Trainer:** This module is invoked when MLAD is required to train for an unknown tuple and it requires human intervention.

#### 3.2.3. Mitigation Phase

This phase is responsible for mitigating the attack and initiating a proper response. The system uses two modules for facilitating mitigation response, i.e., the Actuator module and the Handler module. The Handler component in this module is responsible for executing the mitigation response if flagged by the Actuator module.

(i) Actuator Module
  The Actuator module is responsible for identifying the most suitable mitigation response in the event of an attack within the IoT network. The mitigation response can either send an alarm signal or shut down the communication in the network. When the Actuator module is aware of an appropriate mitigation response, it would activate the Handler module to execute the response or generate an alarm for the end-user.
(ii) Handler Module
  The Handler module is primarily a set of mitigation procedures hard-coded within IID program to execute a mitigation procedure as a proof of concept. A mitigation procedure is invoked by the Actuator module in response to an intrusion, and is further executed by the Handler module. Once the mitigation response is executed successfully by the Handler module, it logs the type of attack and the mitigation response provided. If the Handler module is required to raise an alarm for the user, it flags the discovered intrusion for ’requiring user attention’ and logs this information in the log file.

## 4. Detection Using Deep Learning

In this section, we discuss the detection algorithm in detail. Specifically, we use deep learning, which is a subset of machine learning with increased flexibility and accuracy over classical learning algorithms. We choose deep-learning technique, as it outperforms other solutions in multiple domains that are highly unstructured and form heterogeneous patterns. They also have an advantage over other machine-learning algorithms due to their ability to incrementally learn and extrapolate new features from a limited set of training data. Additionally, the thin and layered structure of sequential deep neural-network models makes them the best fit for being deployed over a low-powered and resource-constrained portable IoT device, still facilitating real-time anomaly detection. Below, we discuss the feature extraction, training, and the traffic classification used in our detection algorithm.

### 4.1. Feature Set

IDSs use behavioral categorization and response to classify malicious and benign communication. No single message, or feature on a communication cycle can determine the behavior or the nature of the communication. Both the qualitative and quantitative features of a communication cycle are required to be observed over a period of time to yield its behavioral characteristics. We propose the features presented in Table 1 to characterize these qualitative and quantitative aspects of wireless communication messages intercepted by the detection module in IoT systems.

In a typical communication cycle between a distinct pair of sender and receiver nodes, the transmission and reception rates are expected to be similar. These values are however different when the system is under an attack such as denial-of-service or sinkhole attack. Similarly, transmission-to-reception ratio is a reasonable indicator of spoofing and masquerading attacks when used in conjunction with activity duration. Transmission mode determines the state and protocol of the communication. Based on the pre-trained behavior, IDS can distinguish if the message headers in a communication cycle have a known vulnerable transmission mode, an unintended sender or receiver (through IP addresses), a malicious payload, or all of these. To classify malicious and benign traffic, the proposed IDS thus gathers above features such as transmission-rate, reception-rate, transmission-to-reception ratio, duration, transmission mode, source-IP, destination-IP, and the data-value information from the network traffic. These features are selected in consideration of the computational capability, and the processing to performance ratio of portable low-powered, resource-constrained IoT devices. Our system caches these features and generates meta-features by preprocessing the data. During data preprocessing, IID calculates the probability distribution of the extracted meta-features as shown in Equation (1).

(1) $$f_{p}=\left\{P\left(B_{0}\right),P\left(B_{1}\right),\ldots ,P\left(B_{7}\right)\right\}$$

where $P(B_{i})$ is the probability of each Byte “1” observed in the $i^{th}$ Byte position, and

(2) $$f=L(f_{0}),$$

where the function $L:R^{8}\mapsto R^{8}$ in Equation (2) is the logical mapping, i.e., if $P(B_{i})$ is greater than a half, the probability is mapped to 1, or else, 0.

The set of features represents a tuple of input data for the machine-learning algorithm. Each tuple is represented as a data-vector $d_{v}$ reduced from *f*, before feeding data into the neural network. Consequently, each meta-feature set can be represented as a feature vector $f_{v}$ at a time instance *n*, generated as,

(3) $$f_{v}\left(n\right)=d_{v}\left(n\right)⊕d_{v}\left(n-1\right),$$

where ⊕ is an exclusive-or operator applied to each position of bits in the vector.

#### Feature Extraction

The proposed IDS uses perceptual learning model for both data collection and feature extraction, as well as for anomaly detection. As described earlier, during the *Network Connection* phase, network traffic is intercepted, and raw features are extracted from the network packets. The data collection and transformation module then produces secondary features, also known as meta-features, by preprocessing the cached primary features. Consequently, the data collection and transformation module concatenates the set of primary features with the set of secondary features to create a tuple. Essentially, each tuple is a set of raw features and the meta-features of a data-packet. Thereafter, the data collection and transformation module feeds the tuple into the perceptual learning model for training.

### 4.2. Deep-Learning-Based Anomaly Detection

**Figure 3:**
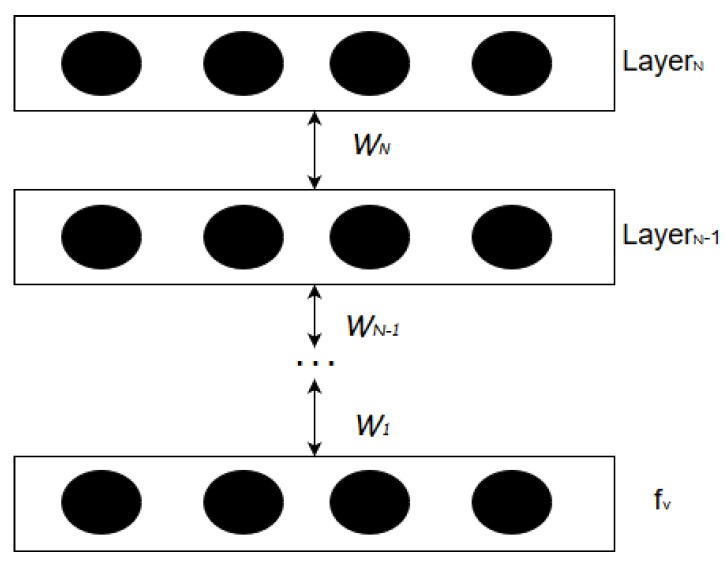
Deep Belief Network Structure.

We use a Deep Belief Network (DBN) to fabricate the feed-forward Deep Neural Network (DNN) as the perceptual learning model. A DBN is a model of un-directed connections between different layers, where each layer comprises *n*-number of neural nodes, while a DNN is a type of feed-forward neural network with many layers. Although a DNN can be fabricated in different ways, an advantage of developing a DNN model from a DBN model is that the DBN layers can be initially trained using unsupervised learning algorithm. DNN can thus be created from a model pre-trained using unsupervised learning which is very fast in comparison to supervised learning. We use the pre-trained layers of DBN model to create a DNN model. (Figure 3).

**Figure 4:**
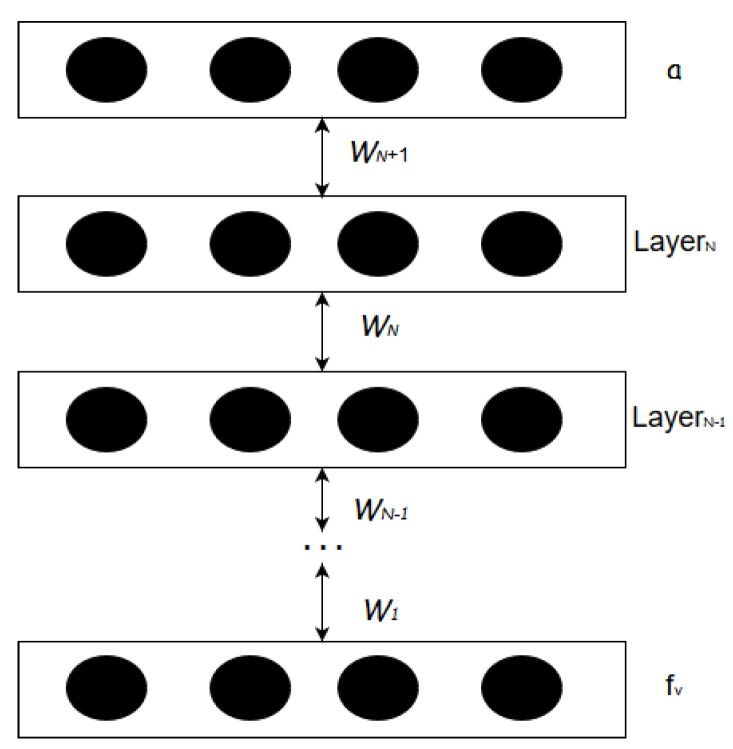
Deep Neural Network Structure.

As shown in Figure 3, the weights for all the hidden layers of this DBN model, denoted by $w_{i}$, are obtained by performing unsupervised training. However, the parameters generated from this unsupervised training are only used for assigning the initial set of weights. For each network transaction, a binary-classification layer and label information (*a*) is added at the top layer of the DBN model to successfully construct a DNN. Figure 4 shows that the DBN is augmented with binary-classification layer and label information to transform into a DNN. Now, this DNN model is trained with a bottom-up supervised learning approach using the label information *a*. During the supervised learning process, each node in a DNN layer is assigned with a weight parameter which are manipulated by using the gradient descent methodology.

The proposed deep-learning model uses supervised training and binary classification for identifying malicious activities. If the DNN detects an unknown anomaly or a zero-day attack, it stores the corresponding tuple of the filtered features to the ‘Cache’ as a feedback. This feedback mechanism is used during retraining of the DNN, which enriches the feature extraction and labeling functionality of the detection system. However, if the extracted features are not sufficient to classify the network traffic, feedback is sent to the data collection and transmission module for retraining.

**Figure 5:**
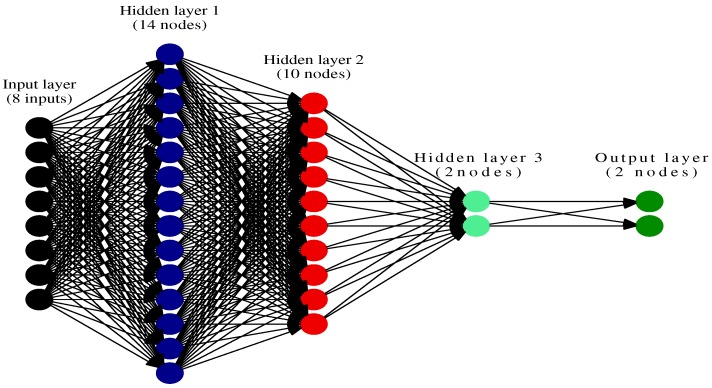
Deep-Learning model for proposed IDS.

As shown in the Figure 5, we developed a 5-layer deep-learning model for this research, containing 1 input layer, 3 hidden perceptual layers, and 1 output layer which is a binary-classifier layer. The input layer comprises of 56 nodes which represents an exhaustive list of *maximum* number of network features that can be fed into the DNN. As mentioned earlier, these input features are represented as a tuple, formed from a combination of both the primary and secondary features. During the supervised training process, each tuple and its label information *a* is fed to the DNN where it passes through the first hidden encode layer and gets filtered out as the *x* most significant features. The *x* features are then passed into the second encode hidden layer where they get filtered into *y* features and the second encode layer feeds them into the third encode hidden layer. The third hidden encode layer takes the *y* features as input from the previous layer and filters two outputs. It also acts as a soft-max layer that fine tunes the results to classify the attack into categories. The result is passed to the output layer representing the classification as malicious and benign traffic. Output layer does not perform any filtration but ingests the output from the third hidden layer and yields the classification result. Thus, the rest of the hidden layers i.e., the second and third encode layers also use the labeled traffic to train themselves in the same way as the first encode layer. Each layer of the DNN thus feeds onto this data, and maps it to a numerical value. The mapped values are normalized to 0 and 1, where benign network traffic is represented by the value 0 and malign network traffic is represented by the value 1. The DNN thus develops a binary classifier for anomaly detection.

As shown in Algorithm 2, the objective function of the proposed DNN model, a *binary_crossentropy* loss function, tries to minimize the total cost in the model (Equation (5)). We retrofit the DNN model for training, and testing the predictions. The proposed IDS is trained and tested against the *testing dataset*. However, if the predictions from testing do not match the results from the *testing dataset*, the system mixes the *training dataset* with the *testing dataset* and re-trains itself with cross-validation.

**Algorithm 2** Intrusion-Detection using Deep-Learning model**Require:** N - List of all header tags from all packets in network interface queue. **function**
Predict(Cache)         /*where cachePipe - is the pipe established with cache*/  matrix←Cache         /*translate packets to matrices*/  Extract features from matrix  Define $dataset_{train}$ & $dataset_{test}$  Initialize Sequential
deep-learning
model  **if**
initialized
**then**   Compilebinary-crossentropy classifier   m←Sequential
deep-learning
model  **end if**  Training: $m\leftarrow dataset_{train}$  **if**
Training is complete **then**   Prediction: $m\leftarrow dataset_{test}$   **if**
Predictions are correct **then**    Re-Train the model   **else**    Invoke Mitigation
Phase   **end if**  **end if**  Store: classi ficationStore←Predictions         /*store the classifier model*/ **end function**

#### Training Deep Neural Network

**Figure 6:**
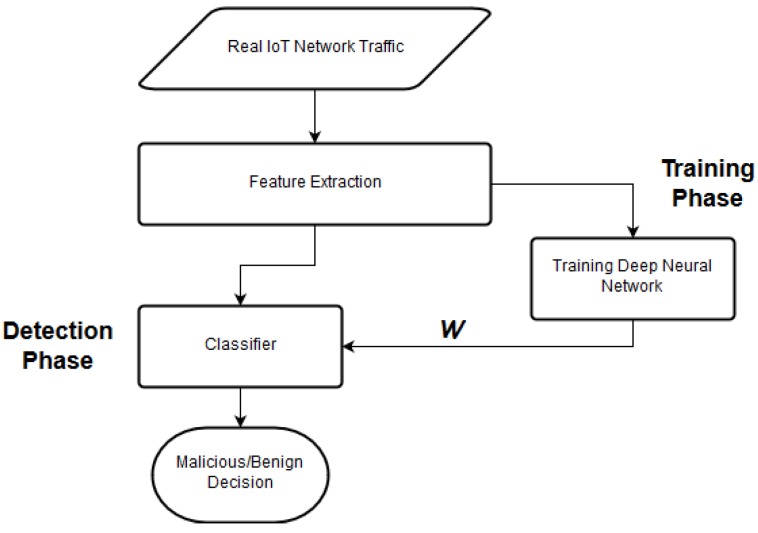
Overview of proposed DNN Training.

Figure 6 details the training mechanism used for the proposed DNN model. At the lowest level, when the feature vector $f_{v}$ is input into the DNN, it passes through each layer of the DNN. Neural nodes in each DNN layer calculates an output using an activation function and generates a filtered result. In this work, we use a rectified linear unit (ReLU) activation function for developing this system. ReLU function is defined as:

(4) f(x)=max(0,x),

with the input x e.g., a matrix from a convolved image. Here, the negative values in the matrix x are set to zero while other values remain constant. Each hidden layer links to the next hidden layer by using linear-combinations of outputs and feeds the filtered output generated by the ReLU activation function to the next layer. To facilitate supervised learning, we fabricate the training set as a set of real-number, *K*, defined as $\left\{\left(f_{v}^{1},a^{1}\right),\left(f_{v}^{2},a^{2}\right),\ldots ,\left(f_{v}^{K},a^{K}\right)\right\}$ samples where each tuple represents a feature vector, $f_{v}^{i}$ and the corresponding binary classification, $a^{i}$. Each feature vector $f_{v}$ represents the probability in the Byte-representation of meta-features generated from a single data-packet, and *a* is the binary label information attached to each data-packet. In the training phase, the input feature $f_{v}$ enters the DNN through the external nodes that are present at the bottom of the DNN. We initialize the weights attached with each neural node in the DNN using the DBN model. Consequently, these weight vectors are modified as more data passes through DNN layers with each cycle in supervised training.

The machine-learning algorithm assigns a cost function, cumulative cost function, and an optimization function [32] to manipulate our detection model. We assign a cost function for each layer of the proposed DNN as formulated in Equation (5), defined as the mean square error function between the prediction value and the output, as,

(5) $$C\left(w,f_{v},a\right)=1\;/\;2{\left\|h_{w}\left(f_{v}\right)-a\right\|}^{2},$$

where *w* is the set of weights designated for each connection between simultaneous layers in the proposed DNN, *a* is the binary label information, and $h_{w}\left(f_{v}\right)$ is the hypothesis function for every meta-feature vector. The hypothesis function $h_{w}\left(f_{v}\right)$ is responsible for manipulating weights *w* on every node in each DNN layer as illustrated in Equation (6), the cumulative cost function for a single set of training data *k*, is defined as,

(6) $$C\left(w\right)=1\;/\;K\sum_{k}C\left(w,f_{v}^{k},a^{k}\right)+\lambda \;/\;2\sum_{n}^{5}\sum_{i}^{M_{l}}\sum_{j}^{M_{l+1}}{\left(w_{ji}^{n}\right)}^{2},$$

where the depth of the DNN model is 5 layers, $M_{l}$ is the number of nodes in the $l^{th}$ layer, and $(w_{ji}^{n})\in w$ are the weights attached to the connection between the $i^{t}h$ node in the layer n−1 and the $j^{t}h$ node in the layer *n*. As mentioned earlier, the ReLU function transforms the weights in the set *w* to generate minimum value for the cost function, $C(w,f_{v}^{k},a^{k})$ and the output of this minimized cost function is assigned to $w^{*}$ as,

(7) $$w^{*}=\left|\underset{w}{\mathrm{minimize}}C\left(w\right)\right|,$$

where *w* denotes the minimum absolute value of the cumulative cost function.

## 5. Implementation and Results

In this section, we demonstrate the implementation of the proposed IDS for IoT networks using a Raspberry Pi. We implement the DNN using Keras, an open-source neural-network library written in Python and test using the open Cooja network simulator developed in Contiki operating system [33]. We also use the Texas Instruments sensor tags CC2650 to create the IoT network testbed and evaluate our results. We use Keras library because of its light-weight, modularity, and easy extensibility, and create a *Sequential* Deep-Learning model, constructed as a linear stack of DNN layers. In addition, Keras library is fast and can process large amounts of data easily. It automatically distributes the work over different processing threads with the machine, without the need for providing optimization or distributed processing parameters as in the case of other machine-learning libraries. Hence, Keras enables implementation of the anomaly-based IDS on a low-powered resource-constrained Raspberry Pi, with a raw processing speed of approximately 700 MHz and a volatile memory of 512 megabytes.

The implementation consists of three stages: input data collection and preprocessing, creation and training of DNN classifier, and testing. Input data collection and preprocessing is used to generate an IoT network-traffic dataset as an input for the anomaly-detection process (ADP). Creation, and training of the DNN classifier are the core sub-processes in the ADP and the detection process in general. Training assigns weights to each classifier node to filter a certain type of input and matures the binary classifier.

### 5.1. Data Preprocessing

The IoT simulation dataset consists of 5 million network transactions (represented as features) from the six sensors distributed in a smart home network simulation. We use Scapy, an open-source network penetration testing framework, to extract these features by stripping down each network packet. The 5 million network transactions were pruned out by the input data-preprocessing program to make the input dataset of 59,529 readings. It is important to note that these network simulations were gathered from two separate simulations, i.e., first simulation with all benign network transactions, and second simulation with a mix of malicious network transactions. Each network transaction in the second network simulation was marked as malicious as the entire network was affected by the malicious activities occurring within the network. In our experimentation, in the dataset of 59,529 transactions, a total of 31,046 network transactions were malicious while the rest of 28,483 network transactions were benign.

### 5.2. Deep-Learning Implementation

As mentioned earlier, we used Python-based Keras machine-learning library for the implementation of the deep-learning algorithm. During classification stage, training dataset are read, stored in a data frame and converted into a matrix. Furthermore, these datasets are bifurcated into the training and testing datasets, where the training dataset comprised of 18,989 benign network transactions and 20,697 malicious network transactions, while the testing dataset comprised of 9494 benign network transactions and 10,349 malicious network transactions. The system was initially tested using the labeled testing dataset consisting of 19,843 (i.e., 33.333% of input dataset) transactions, wherein each record constitutes of the 6 values; transmission-rate, reception-rate, transmission-to-reception ratio, duration, transmission mode, source-IP, destination-IP, the data-value information, and the binary label information. After the initial training of DNN using labeled training dataset comprising of 39,686 (i.e., 66.667% of input dataset) transactions, it was run against the testing dataset, without any binary label information. Since, the testing dataset had 39,686 of unlabeled records, the deep-learning model produces 39,686 predictions in the form of “0” or “1”.

We then create and instantiate a “Sequential” DNN with 3 hidden layers, and equip the processing units within each layer with ReLU activation function. Thereafter, the deep-learning model is compiled and fitted with 150 runs, i.e., epochs, and the number of features. Finally, the deep-learning model is compiled, and the classifier is assigned and saved in the variable “predictions”. Consequently, in every test, the results of the classifier are normalized to a binary value.

### 5.3. Attack Model

We simulate and evaluate the performance of our proposed detection system against various attacks on IoT networks such as the sinkhole attack, distributed denial-of-service (DDoS) attack, blackhole attack, opportunistic service attack and wormhole attack.

- Blackhole Attack: In a blackhole attack, the malicious device falsely advertises shortest route to destination and then silently drops all packets on its path creating a blackhole in the network.
- Opportunistic Service Attack: In an opportunistic service attack, the malicious device increases its trust value by providing highly dependable services at first and then later resorts to providing inferior service for its own profit.
- Distributed Denial-of-Service (DDoS) Attack: In a DDoS attack, multiple compromised IoT devices attack a target server or other network resources resulting in denial of service for users of the targeted resource.
- Sinkhole Attack: In a sinkhole attack, the malicious node may announce beneficial route or falsified path to attract all nodes to redirect their packets through it, acting as a sink.
- Wormhole Attack: In a wormhole tunnel attack, pair of attacker devices collude with each other through a virtual private connection. The network packets received by the victim device is first forwarded through the wormhole, and replayed later, resulting in non-optimized routes.

### 5.4. Evaluation Results

We evaluated the detection system by measuring the performance metrics: recall, precision, and F1 score. Precision (P) and recall (R) are two important metrics used to evaluate detection performance when there is an imbalanced classification, and P-R curve refers to a curve composed of these two metrics. Precision also referred to as the positive predictive value, describes how good a model is at predicting the positive class. Precision can be defined as the ratio of the number of true positives divided by the sum of the true positives and false positives.

(8) $$Precision=\frac{TruePositives}{TruePositives+FalsePositives}$$

Recall is calculated as the ratio of the number of true positives divided by the sum of the true positives and the false negatives.

(9) $$Recall\left(TPR\right)=\frac{TruePositives}{TruePositives+FalseNegatives}$$

F1 score is the weighted harmonic mean of the precision and recall and reflects the balance between P and R.

(10) $$F_{1}=\frac{2*(precision*recall)}{\left(precision+recall\right)}$$

To draw a comparison with existing IDS, we implemented current solution based on inverse weight clustering technique [34] and compared its performance with our proposed detection system. In the following evaluation, DL-Sim refers to the simulation results of the proposed deep-learning (DL)-based IDS, DL Testbed refers to the evaluation results obtained by running deep-learning algorithms on an experimental testbed of IoT devices and IWC refers to the existing IDS solution based on inverse weight clustering. We adopted the same attack data to test the intrusion-detection methods and plotted P-R curves under different attack scenarios. Based on the analysis of P-R curves, we obtain the optimum thresholds and calculate the performance evaluation metrics in different attack scenarios as shown in Table 2, Table 3, Table 4, Table 5 and Table 6.

**Figure 7:**
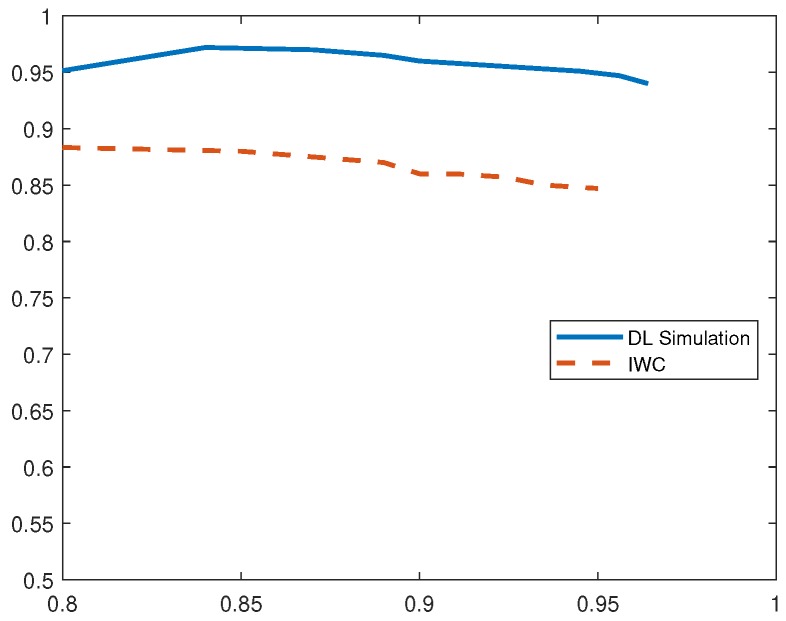
P-R Curves for Blackhole Attack.

From analyzing the P-R curve for blackhole attacks in Figure 7, we observe that the proposed DL-based IDS has an average precision of 97% compared to 89% precision obtained in the related IWC-based IDS solution. The recall value of our system is however comparable to existing solution. We also note that the proposed IDS demonstrates a higher F1 score than other schemes consistently.

**Figure 8:**
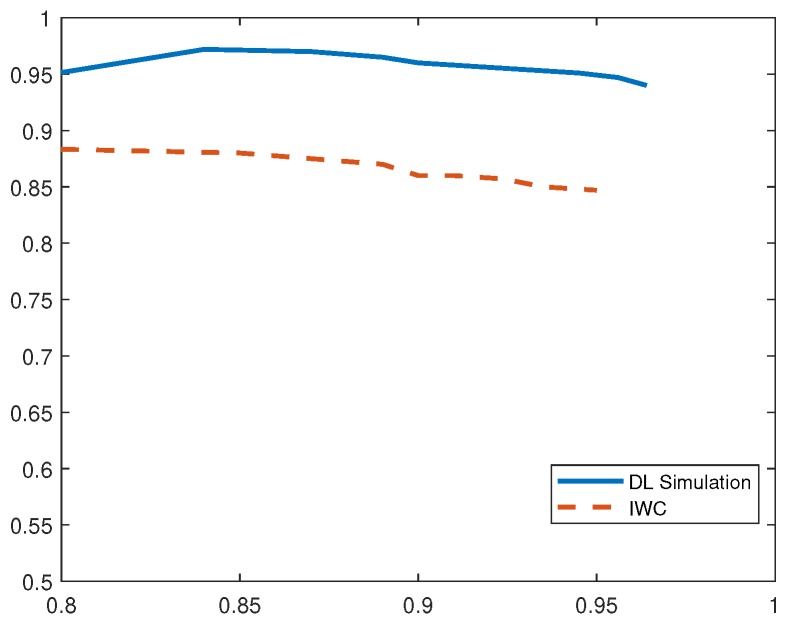
P-R Curves for DDoS Attack.

From Figure 8, we observe that the proposed DL-based IDS demonstrates higher precision (96%) and recall (98.7%) rate for detecting DDoS attacks in comparison to related work with 91% precision and 95% recall rates. Our scheme also presents higher F1 score of 0.973.

**Figure 9:**
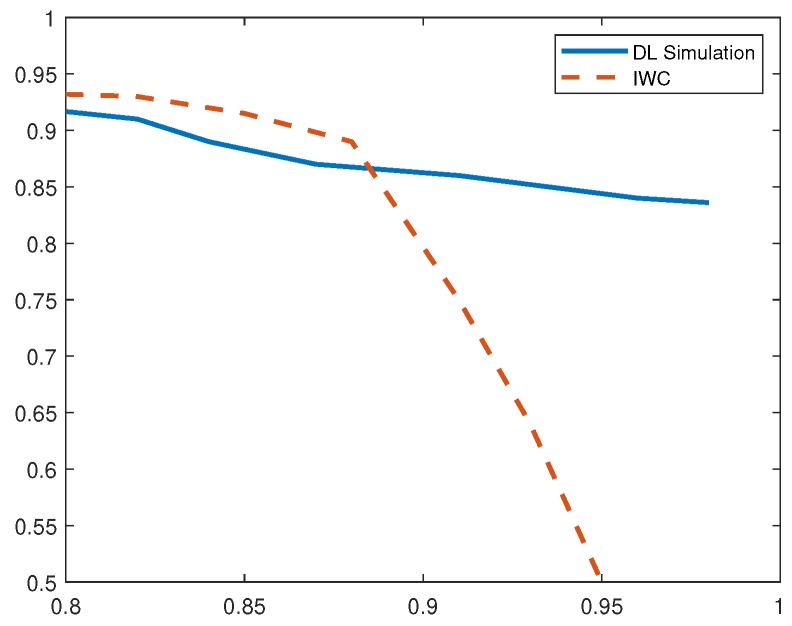
P-R Curves for Opportunistic Service Attack.

Figure 9 shows that the precision for opportunistic service attack normalizes to 95% from the beginning and is slightly lower than the precision value of compared scheme, whereas the recall rates for the systems are comparable. When methods share similar recall values (as presented through TPR), F1-score is an important evaluation metric in determining overall performance. In this case, with comparable TPR values, as F1 score is slightly higher in DL-based IDS, we can conclude that our method performs better.

To establish reliability of the proposed IDS, we further tested the detection system against sinkhole and wormhole attacks on a testbed implementation of IoT networks. Six IoT devices were created using sensor tags and Raspberry Pi functioned as the IID device.

**Figure 10:**
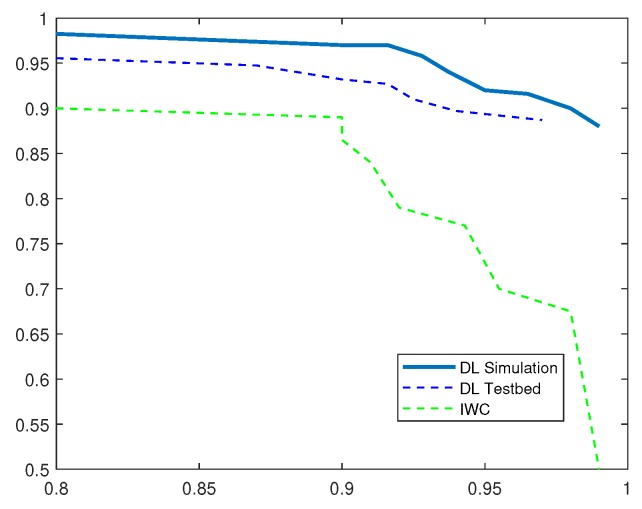
P-R Curves for Sinkhole Attack.

Figure 10 demonstrates high precision value of 99.5% for DL-based IDS through network simulations and a value of 98.47% when implemented on experimental IoT testbed. The recall rate also dropped significantly i.e., from 99% using simulations to 97% while using real sensors. This drop can be attributed to lossy wireless transmission medium in IoT sensor testbed. Despite the drop in recall rate, the F1 score was comparable in both the simulation and testbed implementation of DL-based IDS and higher than the compared system. Both simulation and testbed results seemed to outperform the related scheme in detecting sinkhole attacks.

**Figure 11:**
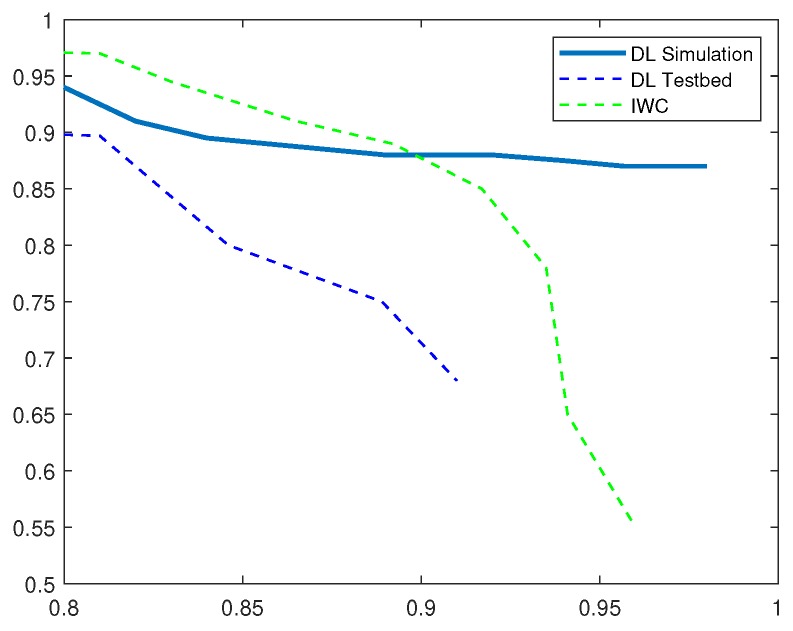
P-R Curves for Wormhole Attack.

Figure 11, presents the precision and recall values for detecting wormhole attacks. We observe that the related detection technique outperforms DL-based IDS with higher precision in both network simulations and experimental testbed scenario. The recall rates are however higher in DL simulation as compared to the IWC detection technique. DL Testbed results show that it does not perform as well as the other simulation-based methods in detecting wormhole attacks. It is important to note that our detection system was trained once before these tests and hence, the training time remains the same. These results validate that the performance of our proposed IDS in real-network setup is comparable to the simulated network traffic. Thus, we can also conclude that our proposed system can robustly detect security attacks under varying network attack scenarios.

## 6. Conclusions and Future Work

In this paper, we investigated the feasibility of deploying machine-learning-based intrusion detection for resource-constrained IoT networks. To that end, we developed an intelligent IDS that tactfully combines network virtualization and DL algorithm to detect anomalous behavior on insecure IoT networks. We investigated the optimal solution for deep-learning-based IDS by evaluating the performance of our scheme against five different attack scenarios, including blackhole attack, opportunistic service attack, DDoS attack, sinkhole, and wormhole attacks. Through analysis of precision-recall curves, we obtained an average precision rate of 95% and recall rate of 97% for different attack scenarios. Our experiments also demonstrate higher F1-scores for all attack scenarios indicating better overall detection performance by the proposed system. Based on the experimentation results obtained from network simulations and testbed implementations, we can conclude that it is both practical and feasible to use DL algorithms for effective anomaly detection in the IoT environment. Future work includes extending the proposed IDS to detect other types of attacks against the IoT including location dependent attacks such as cloning of device ID, spoofing, and sybil attacks. RPL specific misappropriation attacks, isolation attacks, neighbor attacks and direct attacks can also be detected by tracking device IDs and validating journal entries such as in DODAG (Direction-Oriented Directed Acyclic Graph) table. A more distributed and computationally optimized version of IID may be also be used towards identifying zero-day attacks.

## Figures and Tables

**Table 1:** Extracted Feature Set.

*transmission rate*	*reception rate*
*transmission to reception ratio*	*activity duration*
*transmission mode*	*source IP*
*destination IP*	*datavalue in formation*

**Table 2:** Blackhole Attack Detection.

Method	Precision	TPR	F1 Score
DL-Sim	97.2%	96.4%	0.97
IWC	89%	95%	0.92

**Table 3:** Opportunistic Attack Detection.

Method	Precision	TPR	F1 Score
DL-Sim	95.7%	98%	0.97
IWC	94%	98%	0.96

**Table 4:** DDoS Attack Detection.

Method	Precision	TPR	F1 Score
DL	96%	98.7%	0.973
IWC	91%	95%	0.93

**Table 5:** Sinkhole Attack Detection.

Method	Precision	TPR	F1 Score
DL-Sim	99.5%	99%	0.99
DL-Testbed	98.47%	97%	0.97
IWC	98.37%	91.2%	0.94

**Table 6:** Wormhole Attack Detection.

Method	Precision	TPR	F1 Score
DL-Sim	96%	98%	0.97
DL-Testbed	93%	91%	0.92
IWC	98.37%	97%	0.97
